# Second Primary Cancers following Colorectal Cancer in Sicily, Italy

**DOI:** 10.3390/cancers14215204

**Published:** 2022-10-24

**Authors:** Giulia Collatuzzo, Margherita Ferrante, Antonella Ippolito, Alessia Di Prima, Cristina Colarossi, Salvatore Scarpulla, Paolo Boffetta, Salvatore Sciacca

**Affiliations:** 1 Department of Medical and Surgical Sciences, University of Bologna, 40138 Bologna, Italy; 2 Department of Medical, Surgical and Advanced Technologies “G.F. Ingrassia”, University of Catania, Via Santa Sofia 87, 95123 Catania, Italy; 3 Cancer Registry of Catania, Messina, Syracuse and Enna, Via Santa Sofia 87, 95123 Catania, Italy; 4 Mediterranean Institute of Oncology (IOM), Viagrande, 95029 Catania, Italy; 5 Stony Brook Cancer Center, Stony Brook University, Stony Brook, NY 11794, USA

**Keywords:** colorectal cancer, cancer survivors, second primary cancer, cancer mortality, multiple cancers

## Abstract

**Simple Summary:**

This study addressed the under-investigated issue of second primary cancer occurring in colorectal cancer survivors. Our aim was to explore whether patients recovered from a first colorectal cancer were at higher risk of developing a subsequent primary cancer. The hypothesis was that exposure to cancer treatment, enhanced health surveillance and shared risk factors may lead to an excess risk of second primary cancer in this population. The number of cases of second primary cancer exceeded the expected in this population, mainly driven by female genital cancers, and especially observed in the first years after colorectal cancer diagnosis. Our findings are overall consistent with previous studies, providing valuable information to better characterize and predict mortality from second primary cancer in subjects who suffered from first colorectal cancer.

**Abstract:**

Background: Cancer survivors are at risk of developing second primary cancers (SPC). We investigated the risk of SPC in colorectal cancer (CRC) survivors in Sicily, Southern Italy. Methods: We analyzed data from the Eastern Sicily cancer registry covering 2.5 million people diagnosed and followed up between 2003 and 2017. We calculated the standardized incidence ratio (SIR) and 95% confidence interval (CI) of SPC overall and by cancer type, using the general Sicily population rates as reference. Results: A total of 19,040 cases of CRC and 1453 cases of SPC were included in the analysis. Mean age of occurrence of SPC was 68.1. The SIR for any SPC was 1.11 (95% CI 1.05–1.17); it was higher in women (1.18; 95% CI 1.08–1.29) than in men (1.07; 95% CI 0.97–1.14, *p*-value of difference 0.07). The SIR was increased for SPC from the ovary (SIR 2.01; 95% CI 1.33–2.95), kidney (SIR 2.00; 95% CI 1.54–2.56), endometrium (SIR 1.94; 95% CI 1.45–2.54), bladder (SIR 1.22, 95% CI 1.04–1.43) and stomach (1.29; 95% CI 0.98–1.66). The SIR for CRC as SPC was 0.84 (95% CI 0.70–1.01). No increased incidence was found for lung, prostate, breast, thyroid and liver cancer. The SIR for SPC overall and several cancers decreased with time of follow-up. Conclusions: In this population, CRC survivors have an 11% higher risk of developing a SPC than the general population, particularly cancers of the ovary, kidney, endometrium, bladder and stomach. Follow-up for SPC is required, especially during the first 5 years from CRC diagnosis.

## 1. Introduction

Cancer patients are at higher risk of death [1], both because of their specific disease and because of the increased prevalence of comorbidities, including second primary cancers (SPC). The higher occurrence of a new cancer in patients affected by a neoplastic disease has been assessed in multiple studies. Based on their onset before or after 6 months from the first neoplasm, synchronous and metachronous SPC can be distinguished [2]. Possible explanations of the occurrence of SPC are (i) exposure to treatment [3,4], which can enhance the risk of cancer in other organs; (ii) exposure to ionizing radiation due to the tests performed during diagnosis and follow-up [5,6]; and (iii) enhanced surveillance due to the first disease (iv) shared risk factors, both hereditary and acquired, and mechanisms of carcinogenesis underlying both first and SPC (e.g., inflammatory microenvironment, alteration of microbiota, hormonal milieu or immune system) [7,8,9,10,11].

A long duration of survival increases the cumulative risk of developing SPC. Thus, long-term surviving cancer patients are those with the highest risk of SPC [3], and the increase in survival from a cancer diagnosis stresses the need to monitor the occurrence of SPC in these patients. Colorectal cancer (CRC) is becoming one of the health issues of the century, being a predominant neoplasm and given that the age of its occurrence is decreasing dramatically, with increasing incidence in subjects younger than 50 [12]. There is evidence of increased risk of SPC in patients diagnosed with CRC [13]. Kidney and pelvis cancer appears to be the most frequent SPC among colon cancer patients in men, and ovarian and endometrial cancers appear the most in women [13].

The study of the relationship between primary and SPC is important to improving the knowledge of cancer mechanisms and pathways of cancer development [13,14,15], also allowing us to better address the follow-up and monitoring measures of cancer patients [15]. Our aim was to assess the risk of SPC following CRC in a population from Southern Italy, including the identification of the most common types of cancer occurring after primary CRC diagnosis and the quantification of the risk by time after the first CRC, by sex and by anatomical subsites of the first cancer.

## 2. Methods

We analyzed data from the Eastern Sicily cancer registry covering 2.5 million people from four provinces (Catania, Enna, Messina and Siracusa) [16]. The registry is considered to be complete and is included in the Cancer Incidence in Five Continents, the collection of high-quality registries maintained by the International Agency for Research on Cancer [17]. We selected cases of CRC diagnosed between 2003 and 2017 and identified cases of SPC occurring among these patients during the same time period using the unique identification number assigned to all Italian residents [18]. The cancer registry collects data on cancer diagnosed mostly through histology confirmation on the primary tumor, and a minority of cases based on clinical data or imaging. In this population, 91% of cancer cases were defined based on histopathological examination. The remaining cases were defined based on imaging (6%), or a combination of other methods. When focusing on SPC, 98% of the cases included in this analysis had histopathological confirmation. We calculated the number of expected cases of cancer in the cohort of CRC cases based on the incidence rates in the population covered by the registry after adjusting for sex, age group and calendar year. In order to exclude metachronous cancers, we excluded tumors diagnosed during the first six months of follow-up.

We calculated the standardized incidence ratio (SIR) and 95% confidence interval (CI) of SPC overall and by cancer type as the ratio of observed over expected SPC; we also stratified the analyses by site of the first cancer (colon, rectum, and subsites within the colon), sex and time since diagnosis of first neoplasm (<1, 1–4, 4–9 and 10 or more years). We tested heterogeneity and trends in SIR using the chi-square tests proposed by Breslow and Day [19].

In addition to this, we ran two multivariate logistic regression models using SPC as the dependent variable; in the first model, the independent variables included sex, age, chemotherapy (no/yes) and radiotherapy (no/yes); in the second model, a categorical variable was used to combine different possible treatment schemes (neither chemo- or radiotherapy/one among chemo- or radiotherapy/both chemo- and radiotherapy). 

## 3. Results

Between 2003 and 2017, 19,040 CRC cases occurred in the study population. Their distribution by selected characteristics is reported in Table 1. Among them, we identified a total of 1453 cases of SPC, including 937 occurring in men and 516 occurring in women. A total of 968 SPC occurred after a primary colon cancer, whereas 485 SPC occurred after a rectal cancer. The mean age at occurrence of SPC was 68.1 years (67.3 years for SPC after colon cancer and 68.9 years after rectal cancer).

The SIR of any SPC was 1.11 (95% CI 1.05–1.17, Table 2). The following SPC were at increased incidence among CRC survivors: ovarian cancer (SIR 2.01; 95% CI 1.33–2.95, 25 cases), kidney cancer (SIR 2.00; 95% CI 1.54–2.56; 60 cases), endometrial cancer (SIR 1.94; 95% CI 1.45–2.54, 49 cases), bladder cancer (SIR 1.22; 95% CI 1.04–1.43, 147 cases), and stomach cancer (SIR 1.29; 95% CI 0.98–1.66, 56 cases). The only cancer with reduced incidence among CRC survivors was prostate cancer (SIR 0.82; 95% CI 0.68–0.96). The SIR for CRC as SPC was 0.84 (95% CI 0.70–1.01; 120 cases).

The SIR for any SPC after colon cancer was 1.10 (95% CI 1.03–1.17) and that after rectal cancer was 1.13 (95% CI 1.03–1.23; p-heterogeneity 0.6, Table 2). CRC was the only SPC with significantly different SIR according to the site of the first primary cancer (*p*-heterogeneity < 0.001).

The SIR for any SPC in men was 1.07 (95% CI 0.97–1.14), that in women was 1.18 (95% CI 1.08–1.29, p-heterogeneity 0.07, Table 3). CRC and thyroid cancer were the only SPC with a significantly different SIR according to sex (p-heterogeneity 0.005 and 0.01, respectively).

Results of the analysis by time since first primary are reported in Table 4. Results within the first year of follow-up were based on a small number of SPC; an increased SIR was found for liver cancer (3.85; 95% CI 1.68–7.61), kidney cancer (3.29; 95% CI 1.21–7.29) and stomach cancer (95% CI 0.99–4.49). After excluding the first year of follow-up, a decreasing trend in SIR with increasing time since first primary CRC was found for all cancers (SIR 1,18; 95% CI 1.07–1.30; SIR 0.88; 95% CI 0.80–0.95; SIR 0.82; 95% CI 0.75–0.89 for 1–4, 5–9 and 10 or more years since first primary, respectively; *p*-trend < 0.001), A decreasing trend in SIR was also found for lung cancer (*p* < 0.001), stomach cancer (*p* = 0.001), ovarian cancer (*p* = 0.003), CRC (*p* = 0.003), liver cancer (*p* = 0.004), kidney cancer (*p* = 0.01) and bladder cancer (*p* = 0.04). Notably, no trend was detected for ovarian cancer (*p* = 0.9), which was the only SPC with an increased SIR after 10 or more years of follow-up.

Excluding CRC as SPC, the SIR was 1.14 (95% CI 1.08–1.21) in the whole population, 1.16 (95% CI 1.09–1.24) among colon cancer patients, and 1.10 (95% CI 1.00–1.22) among rectal cancer patients (p-heterogeneity 0.4; results not shown in detail).

The results of the multivariate logistic regression models (Table 5) showed a significantly increased risk of SPC in CRC survivors who received chemotherapy (OR = 1.52, 95% CI = 1.35–1.72) but not radiotherapy. A small difference was observed when considering the combination of chemo- and radiotherapy.

## 4. Discussion

Our analysis showed an excess risk of SPC following CRC in an Italian population from four provinces of Sicily. The number of SPC in CRC survivors almost reached 1000 cases, with higher numbers in men in all the provinces despite overall increased risk of SPC in women when pooling the cases of the different cancer types, mainly driven by the high incidence of female genital cancers. Most of the cases occurred in people aged 60 or older, with mean age 67.3. We also observed an association between SPC and chemotherapy but not radiotherapy. The overall higher SIR was, nevertheless, due to few specific cancer types, including ovary, kidney, endometrial and bladder cancer, and stomach cancer with borderline significant increase. On the contrary, prostate cancer resulted in reduced in CRC survivors. No statistically significant risk was found for lung, breast, thyroid and liver cancer. We observed a 10% increased risk of any SPC after colon cancer, and 13% after rectal cancer, with CRC being the only SPC with significantly different SIR based on the site of the FPC. We found a significant 18% increased risk of SPC in the first 5 years from CRC diagnosis, and an overall significant decreasing trend in the risk of SPC with time. Endometrial cancer was an exception, showing a persistent higher risk of occurrence up to 10+ years from FPC.

The overall SIR we found is consistent with that calculated in recent articles. For example, another population-based study based on 189,890 colon and 83,802 rectal cancer cases from the Surveillance, Epidemiology and End Results Program (SEER) database reported a 13% increased SIR (1.12–1.15) overall, similar to our findings [20]. A monography focused on the incidence of new malignancies in cancer survivors has been built based on data collected between 1973 and 2000 in the SEER Cancer Registries [7]. The authors reported a significant 14% increased risk of SPC in all ages, with the younger the age the higher the proportion of excess observed cases than the expected, 6 folded in the pediatric age up to a 2% increased risk in 70–79 years old people [7]. Consistent with our findings, they reported a higher risk of cancer in the first 5 years after initial diagnosis, and explained this as the consequence of more strict monitoring in the very first period compared to the following, and possibly also as a result of a change in behavioral patterns [7].

Age at the onset of the FPC was implied in the following risk, as the likelihood of cancer in survivors was not higher than in the general population in individuals diagnosed with CRC when older than 70 years. The RR of all new cancers combined was slightly higher in females and in Blacks. Also, the follow-up period showed different range of risk, as 10% of the excess risk was found in the first 5 years, while it became close to normal levels for later follow-up intervals, which is consistent with our findings [7]. The risks of subsequent malignancies were significantly increased for cancers of the tongue, oropharynx, stomach, small intestine, colon, rectum, anus, bile ducts, uterine corpus, kidney, and ureter. In contrast, lower risks were seen for cancers of the lip, larynx, lung, liver, and gallbladder, as well as for chronic lymphocytic leukemia. Despite some discrepancies, the direction of these results is somewhat consistent with what we observed in our study population, where for example no excess risk or even a decreased number of cases than the expected were found for lung and liver cancers [7].

A large population-based cohort study from Taiwan including almost 100,000 CRC patients over 16 years showed a significantly higher risk of ovary (SIR = 2.00, 05% ci = 1.52–2.57), uterus (SIR = 3.20, 95% CI = 2.62–3.87), thyroid (SIR = 1.71, 95% CI = 1.34–2.1), kidney (SI = 1.45, 95% CI = 1.25–1.67), bones and soft tissues (SIR = 1.38, 95% CI = 1.01–1.85), bladder (SIR = 1.31, 95% CI = 1.16–1.48), breasts (SIR = 1.20, 95% CI = 1.06–1.35), prostate (SIR = 1.19, 95% CI = 1.09–1.31), and lungs and mediastinum (SIR = 1.18, 95% CI = 1.10–1.26) compared to the general population [21]. While the association found with ovary, uterus, bladder and kidney agree with our results, those for breast and lung are discrepant, and the results for prostate cancer are in contrast with our findings. The median age at diagnosis of SPC was 67, which is approximately the same that we found. The authors calculated an overall 13% excess risk of occurrence of SPC after CRC diagnosis and a median time of development of 4.7 years. Interestingly, the younger the age, the higher the cumulative risk, which became not significantly different from the general population among women aged ≥ 80 [21].

When comparing our cancer-specific results with the SEER database [20], few differences can be demonstrated: higher risk was reported for stomach, endometrium and kidney cancers, with similar magnitude to that which we described; regardless, contrarily to our results, an increased risk was also found for lung, prostate and thyroid cancers, while bladder cancer did not significantly increase. The main discrepancy between our analysis and the SEER analysis concerns ovary cancer, which was shown to be less likely to occur in CRC survivors while being one of the strongest associations we found. The SEER database also showed that patients in the youngest range age (20–39) had the highest risk of any SPC [20].

Some types of SPC are known to occur more frequently in CRC survivors than in the general population. The strong association between CRC and ovarian cancer is explained by the evidence of common genetic factors such as hereditary non-polyposis colorectal cancer (HNPCC) [22].

Our findings agree with a large study conducted on the Thames Cancer Registry in England between 1961 and 1995, where a 2.56 SIR and a 1.59 SIR were reported for ovary cancer in women with a previous diagnosis of CRC before 65 years old and ≥65 years old, respectively [23]. Also, the study specified that this increased proportion was limited to the first five years after CRC [23]. This study also pointed out that HNPCC alone could not explain the excess number of ovary cancer cases in subjects with previous CRC, suggesting the implication of additional factors. The short latency period among the two cancer diagnoses may be related to the misclassification of ovary cancer as primary instead of a metastasis from CRC [23]. Indeed, 3–5% of CRC patients develop ovarian metastases [24]. Noticeably, recent surgeons’ classification of metastases distinguishes early (≤12 months of diagnosis from primary disease) and late (>12 months from diagnosis) metachronous metastases [25]. Thus, based on the criteria for the assessment and definition of synchronous and metachronous metastases, the SIR of SPC deeply varies. Besides this, as the SIR for ovarian cancer after CRC primary cancer remains high in the following calendar years periods, an overall increased risk of developing ovarian cancer in women with primary CRC is suggested. The heritable syndrome HNPCC predisposes not only to multicentric cancers of the colon and rectum, but also to early-onset cancers of the small intestine, stomach, bile ducts, uterine corpus, ovary, renal pelvis/ureter, and brain [26]. Similarly, the extra- colonic tumors associated with FAP syndrome include the stomach and small intestine (duodenal and periampullary) in addition to thyroid and brain tumors [27]. These syndromes and their variants may be implicated in the notably high risks for subsequent tumors of diverse sites among younger patients (ages < 40 years) with colon cancer, although excess risks were also seen to a limited extent among older persons. The role of genetic susceptibility suggesting HNPCC is also evident in the reciprocally increased risks for colon cancer following cancers of the uterine corpus, ovary, and renal pelvis/ureter diagnosed at young ages. It should be noted that hereditary syndromes can be only partially expressed at the phenotypic level [28].

Our analysis found an overall increase of the risk of stomach cancer occurring after CRC diagnosis. This is consistent with other studies. Robertson and coworkers assessed a pooled SIR of 1.22 for stomach cancer (95% CI = 1.07–1.39; *p* = 0.003) in their review [29]. We found a higher magnitude of this association, despite the small sample size prevent us from a full comparison with Robertson’s data. Indeed, when considering the single provinces, a significant higher SIR was found only for one province (Siracusa). A hypothesis is that in this area there are shared risk factors, such as genetics, which enhance the odds of occurrence of gastric cancer in CRC patients. Indeed, if on the one hand gastric cancer is not largely due to genetics, on the other the cases we described are second primary, then possibly subjected of higher genetic pressure. Next, a higher prevalence in Hp, which is known to vary by geographical area and populations, may play a role in the pattern of distribution of second primary gastric cases in Sicily [30].

We found higher kidney and bladder cancer cases numbers than expected. This is consistent with Robertson’s review, published in early 2022, which showed a pooled SIR equal to 1.19 (95% CI 1.06–1.33; *p* = 0.003) for bladder and to 1.50 (95% CI = 1.19–1.89; *p* = 0.0007) for kidney cancer [29]. Our results are close to these values, and the limited significance probably reflect the small sample size we observed. Indeed, we may have caught the positive direction of second primary bladder cancers following CRC which have been identified by the review in a very large population (7,716,750) based on seven studies. While a common exposure to environmental risk factors may be hypothesized, we would have observed a higher risk of other endodermal-derived epithelial cancers such as lung cancer, according to the idea that similar susceptibility and response to environmental factors and subsequent mutations. In the same way, smoke is unlikely to be related to this pattern of increase [21]. As noticed by Robertson et al., a possibility is that neoplasms such as ovary, stomach and bladder ones may be detected during the chest and abdominal computer tomography (CT) scan, contributing to a higher detection rate [29].

Our analysis showed a decreasing trend in the risk of SPC occurrence in the years from FPC of colorectum. This suggests that the excess risk of SPC is not due to common risk factors but rather to the effect of cancer therapies. When looking at Table 4, however, some exceptions can be noticed: breast and prostate cancer showed no trend, consistent with the fact that they did not turn out to be associated to CRC as FPC; thyroid cancer also lacks a significant trend, likely due to the small number of cases included in the study; endometrial cancer was the only cancer type which showed to be at an increased risk with a constant trend in time. This last case could be explained by the presence of common risk factors between CRC and endometrial cancer (e.g., body max index (BMI), Lynch syndrome). Moreover, the consistent evidence of increased number of SPC diagnosed within the first year after FPC which can be found in literature and which we also observed may be due to a higher attention to cancer surveillance at the time and shortly after the diagnosis of FPC in the first period, which was progressively reduced during follow-up. If indeed there is increased surveillance in the first period, cancers that would normally be diagnosed later would have their diagnosis anticipated, and the apparent excess in the first period of follow-up would be (at least in part) the result of this phenomenon.

Time of occurrence is an important aspect to take into consideration when analyzing SPC epidemiologic and clinical characteristics. For example, a worse profile was described in endometrial SPC that occurred 10+ years from the first cancer diagnosis [31]. Based on the time-definitions, different conclusions can be obtained around the calendar-year analyses currently available. For cancers which have medium-long survival time, such as CRC, six months are considered reasonable to distinguish metachronous from synchronous SPC [32]. Such results highlight the importance that CRC survivors should be monitored to detect SPC development within 1 year. To this regard, machine learning can help in the prediction of SPC [33] and also in distinguishing it from recurrence [34]. Moreover, SPC themselves can promote the recurrence of the FPC [35].

When focusing on the different anatomical sites of FPC, no significant difference can be observed in the occurrence of SPC after colon and rectum cancer, except for colorectum SPC; as illustrated in Table 2, rectal cancer is significantly associated with a risk of following CRC, while colon cancer is not. This may be due to the different therapeutic approach to the two cancer sites, where rectal cancer requires surgical resection of the rectum, preventing the following development of the cancer in the same site, while colon cancer usually implies partial resection or radio/chemotherapies [36]. Indeed, tumor progression after partial resection has been described as a consequence of the phenomenon of the “compensatory hyperplasia” [36], and more recently the hypothesis of surgery-induced tumor growth based on hypoxia and inflammation of the resected tissues has been discussed [37].

Small differences were observed in SPC occurrence in men and women CRC survivors. As presented in Table 3, the only significant difference regards the proportion of second primary CRC, which occurs more often in men than in women, despite the fact that the risk is not significantly increased in men per se. Similarly, a gender difference can be seen in thyroid cancer, which, however, is impaired by a small number of subjects, as previously mentioned. Overall, women turned out to be more likely to develop a SPC of any type compared to men. This is explained by the number of endometrial and ovary cancers, which are important female genital cancers. On the contrary, cancers of the male genitals (such as prostate cancer) are not increased in CRC survivors.

The investigation of SPC asks for attention and effort. An interesting question is the determination of the risk of the same cancer in individuals with a family history of a certain SPC [38]. Indeed, the multiple primary tumors found in CRC may be associated with hereditary CRC and could be explained by genetic profiling [39]. For example, the occurrence of first CRC followed by second CRC have been commonly identified with *BRCA1/BRCA2*, and *MMR* genes [40].

The combination of certain cancer sites can be linked to a specific syndrome. In these cases, the pattern of multiple primary tumors is highly specific, indicating that they are almost certainly of genetic origin, like colon and endometrial cancer in Lynch syndrome [39]. Besides this, some multiple primary cancers are known to be statistically associated but the genetic basis is unknown (such as in the case of stomach and kidney cancers).

Cigarette smoking and alcohol use were indicated as common risk factors explaining the occurrence of multiple cancers, corresponding to the cause of >35% of the total excess found by the SEER survey [7]. It seems that the same strategies aimed at reducing the risk of first primary cancers may be useful to reduce the risk of new malignancies in survivors (e.g., diet, physical activity, no smoking, reducing alcohol consumption). As noted by the authors, the challenge is in identifying low-penetrant gene polymorphisms involved in modifying the carcinogenic risks of lifestyle, environmental and treatment-related exposures [7].

With regard to cancer treatment as a risk factor for subsequent SPC development, we observed a role of chemotherapy as an independent risk factor for SPC, which is consistent with several studies on different cancer types [2,3], despite the fact that no significant relationship was found between overall SPC in CRC survivors and radiotherapy. The lack of an independent effect of radiotherapy on the risk of SPC in this population is possibly due to the relatively short follow-up.

Based on SEER data, the overall risk of developing an SPC after colon cancer was 7%, vanishing when excluding subsequent cancers of the colon, rectum, and anus [7]. Conversely, the excess SIR we found for total SPC was even higher when excluding these cancer types from our analysis. This may reflect a different pattern of risk in this study population. This study has several strengths. First, we addressed a relevant medical issue, namely SPC, which is emerging in the daily practice of oncology. This is one of the few studies on SPC from Italy, and the first on SPC occurrence in CRC survivors on a population from Southern Italy. The population-based design is a point of strength; indeed we analyzed a large population from Sicily Cancer Register, collecting accurate sociodemographic and cancer-related data. We performed a number of analyses and were able to show stratified results. Furthermore, we included in our analysis a high number of types of SPC. Moreover, given the single source of data for this study, the cut-off for the definition of metachronous and synchronous cancer is standardized. Our results are for the most part consistent with the previous literature, and therefore are likely to be solid.

This study also has some limitations. First, the analysis lacks adjustment for confounding factors which may have influenced the results (e.g., cigarette smoking, BMI, diet and alcohol). With regard to dietary factors, this population is likely to follow a Mediterranean diet, rich in fish and vegetables and fruit and with small amount of meat intake [41,42]. Family history of cancer was missing, preventing additional insight on the possible clustering of cancer cases within family groups. Regardless, this is common for analyses based on cancer registries, given that the information does not include individual characteristics which may be cancer risk factors. Also, while on the one hand we were able to perform several stratified analyses, on the other their statistical power was low because of the small sample size. For the same reason, the statistical significance of some results may be impaired by the multiple comparison issue. In addition, the relatively short follow-up could have limited the observation of SPC in this population. Indeed, we cannot distinguish whether the increased number of SPC observed in this population was due to the enhanced monitoring of CRC survivors, leading to an anticipation of cancer diagnoses which would have been identified later, to a genuine increase in risk, or to both. Thus, many results should be read with caution. Furthermore, the results we obtained are likely not to be fully generalizable. Despite this, given the specificity of the topic we addressed, it is reasonable that the associations we found are suggestive of a pattern of risk which can indeed be found in other populations. The consistency of our results with the previous literature supports this possibility.

## 5. Conclusions

In conclusion, this study provided data on the prevalence and the characteristics of SPC following CRC in a population from Southern Italy. The increased risk of SPC in CRC survivors is mainly due to the higher number of certain types of cancer, including ovary, kidney, endometrium, bladder and stomach, compared to the general population. These increased risks mainly referred to the first 5 years of follow-up. Liver, prostate and lung cancer showed to be less likely to occur in CRC survivors in the long-term compared than the general population. Our results suggest that the occurrence of SPC, other than for the colon and rectum, should be considered in CRC patients, who therefore need specific checks. In particular, the monitoring of CRC survivors should cover the first 5 years of follow-up. Further studies considering additional epidemiological data such as lifestyle risk factors and family history of cancer would be needed to better interpret these findings.

## Figures and Tables

**Table 1:** Distribution of cases of CRC and of SPC, by selected characteristics.

	CRC Primary Cancer		SPC		
	N	%	N	%	Chi2
**Total**	19,040	100.0	1453	100.0	
**Sex**					*p* < 0.001
Male	10,039	52.7	937	64.5	
Female	9001	47.3	516	35.5	
**Age (years)**					*p* < 0.001
<50	1182	6.2	71	4.9	
50–59	2548	13.4	160	11.0	
60–69	4547	23.9	446	30.7	
70–79	6127	32.2	562	38.7	
≥80	4636	24.3	214	14.7	
**Calendar year**					*p* < 0.001
2003–2007	5960	31.3	700	48.2	
2008–2012	6475	34.0	504	34.7	
2013–2017	6605	34.7	249	17.1	
Anatomical site *					*p* = 0.3
Colon	12,937	67.9	968	66.6	
Rectum	6103	32.1	485	33.4	
**Treatment**					*p* < 0.001
No CT/RT	10,712	60.9	731	50.3	
CT/RT	6,343	36.1	662	45.6	
CT&RT	532	3.02	60	4.13	

CRC, colorectal cancer; SPC, second primary cancer; CT = chemotherapy; RT = radiotherapy; CT/RT = chemotherapy or radiotherapy; CT&RT = chemotherapy and radiotherapy. * The anatomical site refers to the specific organ from which the first primary cancer occurred, and that from which SPC occurred.

**Table 2:** SIR of selected SPC following CRC, 2003–2017.

SPC	Colon			Rectum			CRC			p het
	Obs	SIR	95% CI	Obs	SIR	95% CI	Obs	SIR	95% CI	
All cancers	968	1.10	1.03–1.17	485	1.13	1.03–1.23	1453	1.11	1.05–1.17	0.6
Stomach	42	1.42	1.04–1.90	14	1.02	0.57–1.66	56	1.29	0.98–1.66	0.3
CRC	43	0.35	0.26–0.47	76	1.29	1.02–1.60	120	0.84	0.70–1.01	<0.001
Liver	33	1.20	0.84–1.67	8	0.64	0.29–1.21	41	1.02	0.74–1.37	0.1
Lung	85	0.87	0.70–1.07	45	0.98	0.72–1.30	130	0.91	0.75–1.07	0.5
Breast	74	0.89	0.72–1.13	32	0.94	0.65–1.32	106	0.92	0.75–1.11	0.8
Endometrium	35	1.96	1.38–2.69	14	1.87	1.06–3.06	49	1.94	1.45–2.54	0.9
Ovary	20	2.34	1.58–2.57	5	1.28	0.46–2.84	25	2.01	1.33–2.95	0.2
Kidney	43	2.12	1.55–2.83	17	1.75	1.05–2.74	60	2.00	1.54–2.56	0.5
Bladder	99	1.21	0.99–1.46	48	1.25	0.93–1.64	147	1.22	1.04–1.43	0.2
Prostate	96	0.84	0.69–1.03	42	0.74	0.54–1.00	138	0.82	0.68–0.96	0.5
Thyroid	15	0.98	0.57–1.57	7	0.86	0.37–1.72	22	0.94	0.60–1.39	0.8

SIR, standardized incidence ratio; SPC, second primary cancer; CRC, colorectal cancer; Obs, observed; CI, confidence interval; p het, *p*-value of test of heterogeneity of SIR between colon and rectum.

**Table 3:** SIR of selected SPC following CRC, by gender (only CRC as first primary).

SPC	Men			Women			p het
	Obs	SIR	95% CI	Obs	SIR	95% CI	
All cancers	937	1.07	0.97–1.14	516	1.18	1.08–1.29	0.07
Stomach	36	1.11	0.79–1.52	20	1.42	0.89–2.15	0.4
CRC	78	1.05	0.83–1.30	42	0.62	0.45–0.83	0.005
Liver	27	0.84	0.56–1.21	14	1.22	0.69–2.00	0.3
Lung	107	0.76	0.62–0.91	22	0.90	0.57–1.34	0.5
Breast	1	-	-	105	1.52	1.25–1.83	-
Endometrium	-	-	-	49	1.94	1.45–2.54	-
Ovary	-	-	-	25	2.01	1.33–2.95	-
Kidney	42	1.75	1.28–2.34	18	2.12	1.29–3.28	0.5
Bladder	127	1.02	0.85–1.21	21	1.30	0.82–1.95	0.3
Prostate	138	0.82	0.68–0.96	-	-	-	-
Thyroid	13	1.77	0.98–2.95	9	0.62	0.30–1.13	0.01

SIR, standardized incidence ratio; SPC, second primary cancer; CRC, colorectal cancer; Obs, observed; CI, confidence interval; p het, *p*-value of test for heterogeneity of SIR between men and women.

**Table 4:** SIR of selected SPC following CRC, by time since first primary CRC.

SPC	<1 year			1–4 years			5–9 years			10+ years			Trend
	Obs	SIR	95% CI	Obs	SIR	95% CI	Obs	SIR	95% CI	Obs	SIR	95% CI	
All cancers	91	0.91	0.73–1.11	399	1.18	1.07–1.30	481	0.88	0.80–0.95	482	0.82	0.75–0.89	<0.001
Stomach	7	2.27	0.99–4.49	24	2.34	1.53–3.42	15	1.06	0.62–1.72	10	0.71	0.36–1.26	0.001
CRC	15	0.7	0.27–0.76	37	0.46	0.33–0.63	40	0.28	1.20–0.38	28	0.22	0.15–0.32	0.003
Liver	7	3.85	1.68–7.61	13	1.46	0.81–2.44	16	1.19	0.70–1.88	5	0.35	0.11–0.67	0.004
Lung	9	1.15	0.55–2.10	50	1.63	1.22–2.13	26	0.54	0.35–0.77	28	0.49	0.33–0.70	<0.001
Breast	2	0.30	0.05–1.01	19	0.87	0.54–1.33	32	0.94	0.65–1.30	53	1.03	0.77–1.33	0.5
Endometrium	2	1.71	0.28–5.64	11	2.39	1.25–4.14	12	1.55	0.83–2.63	24	2.05	1.34–3.01	0.9
Ovary	2	2.89	0.50–9.86	10	3.95	2.01–7.04	8	1.93	0.89–3.67	5	0.85	0.31–1.90	0.003
Kidney	5	3.29	1.21–7.29	18	2.93	1.79–4.54	22	2.19	1.41–3.26	15	1.22	0.71–1.97	0.01
Bladder	8	1.11	0.51–2.10	48	1.75	1.30–2.31	42	1.03	0.75–1.38	51	1.12	0.84–1.45	0.04
Prostate	3	0.36	0.09–0.98	28	0.90	0.61–1.28	48	0.97	0.72–1.28	59	1.15	0.88–1.47	0.3
Thyroid	0	0	0–6.37	4	1.02	0.32–2.46	5	0.71	0.26–1.57	10	0.75	0.38–1.34	0.7

SIR, standardized incidence ratio; SPC, second primary cancer; CRC, colorectal cancer; Obs, observed; CI, confidence interval. Trend, *p*-value of test for linear trend, excluding the category < 1 year.

**Table 5:** Risk of SPC in CRC survivors by type of treatment of CRC—results of the multivariate analysis.

Treatment	OR, 95% CI	*p* Value
Chemotherapy		
- No	Ref	
- Yes	1.52, 1.35–1.72	<0.001
Radiotherapy		
- No	Ref	
- Yes	1.12, 0.86–1.47	0.412
Therapy scheme		
- No CT/RT	Ref	
- CT/RT	1.54, 1.37–1.75	<0.001
- CT&RT	1.63, 1.23–2.16	0.001

Notes: OR = odds ratio, adjusted for sex and age; CI = confidence interval; CT = chemotherapy; RT = radiotherapy; CT/RT = chemotherapy or radiotherapy; CT&RT = chemotherapy and radiotherapy.

## Data Availability

Data can be provided to external investigators upon reasonable request and agreement of the Institutions involved.
